# Extent of late gadolinium enhancement in patients with hypertrophic cardiomyopathy in correlation with serum MMP9 as an indicator of myocardial fibrosis

**DOI:** 10.1186/1532-429X-16-S1-P291

**Published:** 2014-01-16

**Authors:** Maxim Avanesov, Monica Patten, Julia Münch, Dennis Säring, Peter Bannas, Enver Tahir, Gerhard Adam, Gunnar Lund

**Affiliations:** 1 Center for Radiology and Endoscopy, Diagnostic and Interventional Radiology, Hamburg, Germany; 2 University Heart Center, General and Interventional Cardiology, Hamburg, Germany; 3 Center for Experimental Medicine, Computational Neuroscience, Hamburg, Germany

## Background

Myocardial fibrosis is known to be associated with abnormal cardiac remodeling and a poorer prognosis in patients with hypertrophic cardiomyopathy. Our purpose was to compare the size of late gadolinium enhancement (LGE) obtained by cardiac magnetic resonance (CMR) imaging in patients with hypertrophic cardiomyopathy (HCM) with serum MMP9, which is a marker of myocardial fibrosis.

## Methods

CMR was performed in 50 patients with HCM (mean age: 54.9 ± 14.1 years, 27 women) using a 1.5 Tesla scanner (Achieva, Philips). Size of fibrosis was quantified in percent of total myocardium on inversion-recovery images after injection of 0,2 ml/kg gadolinium using the HeAT software and compared with levels of serum MMP9. A serum level of > 46 ng/ml was regarded as increased.

## Results

Nine out of 50 patients with HCM (18 %) showed no fibrosis on LGE-CMR. In the remaining 41 patients, mean size of fibrosis was 13,3 ± 10,3% on LGE-CMR. In all patients, the mean MMP9 level was 54,4 ± 35,2 ng/ml. Size of fibrosis on LGE-CMR strongly correlated with MMP9 levels (R2 = 0,557, Pearson's r = 0,75, p < 0,01). In the 9 patients with no LGE, MMP9 was with 29,6 ± 14,2 ng/ml significantly lower compared to the 41 patients with LGE and levels of 59,8 ± 36,2 ng/ml (p = 0,01).

## Conclusions

Size of fibrosis on LGE-CMR strongly correlated with the serum fibrosis marker MMP9 in patients with HCM. There were no patients with increased fibrosis on LGE-CMR and normal MMP9, so that an increased MMP9 makes a myocardial fibroses observed by LGE-CMR probable. However, 2% of patients revealed no LGE despite increased MMP9 levels. These patients may have a diffuse myocardial fibrosis which is not detectable by standard LGE-CMR.

## Funding

No Funding has been paid during the study.

**Figure 1:**
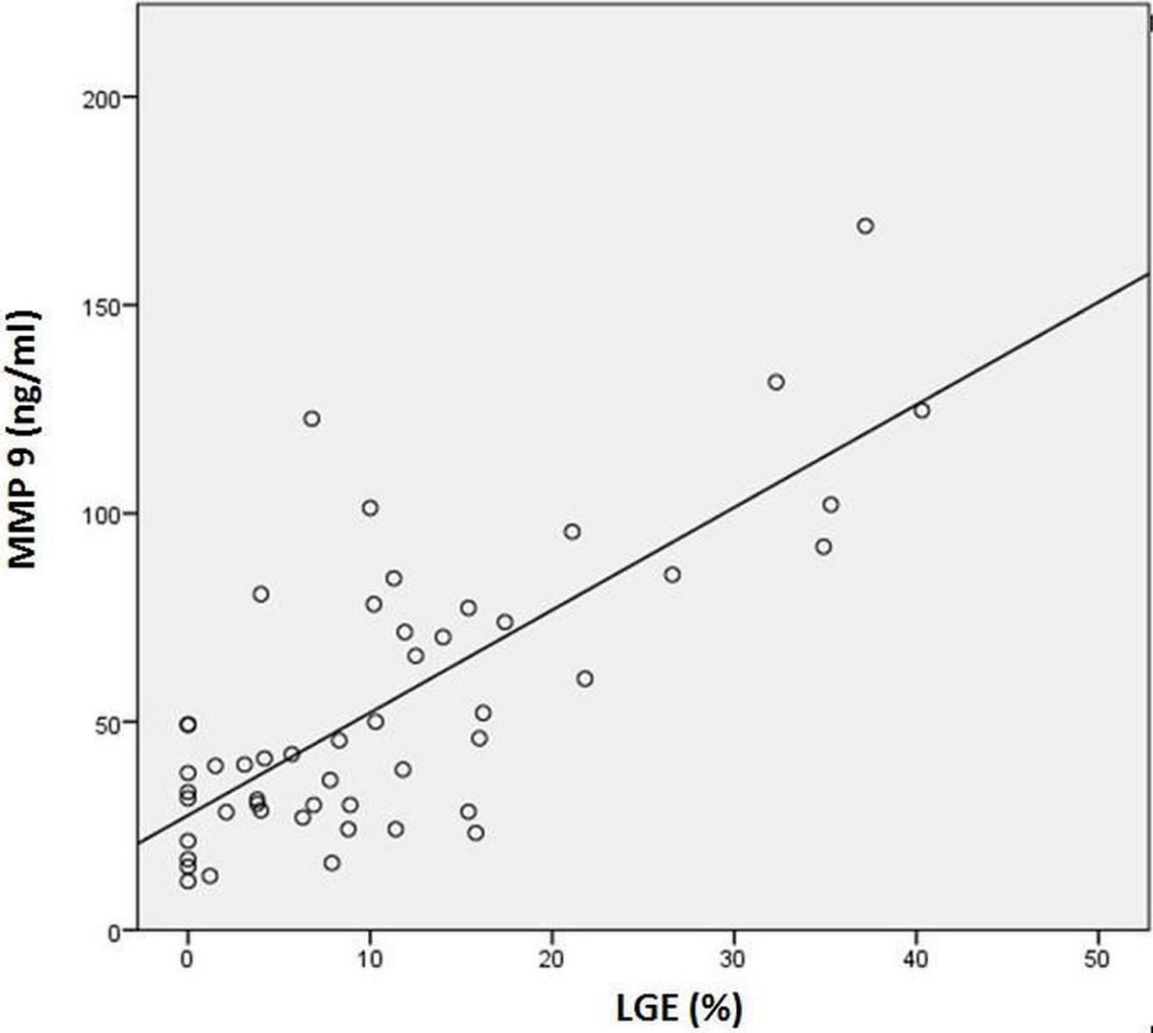
**Correlation between LGE and MMP 9values (Pearson's r = 0,75, p < 0,01)**.

